# An Evaluation of the Replicable Factor Analytic Solutions Algorithm for Variable Selection: A Simulation Study

**DOI:** 10.1177/00131644251377381

**Published:** 2025-11-03

**Authors:** Daniel A. Sass, Michael A. Sanchez

**Affiliations:** 1 University of Texas at San Antonio, USA; 2 H-E-B and University of Texas at San Antonio, USA

**Keywords:** factor analysis, variable selection, replicability, RFAS algorithm

## Abstract

Observed variable and factor selection are critical components of factor analysis, particularly when the optimal subset of observed variables and the number of factors are unknown and results cannot be replicated across studies. The Replicable Factor Analytic Solutions (RFAS) algorithm was developed to assess the replicability of factor structures—both in terms of the number of factors and the variables retained—while identifying the “best” or most replicable solutions according to predefined criteria. This study evaluated RFAS performance across 54 experimental conditions that varied in model complexity (six-factor models), interfactor correlations (ρ = 0, .30, and .60), and sample sizes (*n* = 300, 500, and 1000). Under default settings, RFAS generally performed well and demonstrated its utility in producing replicable factor structures. However, performance declined with highly correlated factors, smaller sample sizes, and more complex models. RFAS was also compared to four alternative variable selection methods: Ant Colony Optimization (ACO), Weighted Group Least Absolute Shrinkage and Selection Operator (LASSO), and stepwise procedures based on target Tucker–Lewis Index (TLI) and ΔTLI criteria. Stepwise and LASSO methods were largely ineffective at eliminating problematic variables under the studied conditions. In contrast, both RFAS and ACO successfully removed variables as intended, although the resulting factor structures often differed substantially between the two approaches. As with other variable selection methods, refining algorithmic criteria may be necessary to further enhance model performance.

Exploratory Factor Analysis (EFA) is a multivariate, data-driven technique used to uncover the underlying factor structure of a set of observed variables and is essential for developing and validating measurement instruments. One of EFA’s key objectives is to model the correlations among observed variables using a smaller number of latent variables (factors). When working with new measures or instruments, researchers often lack prior knowledge about the number of factors or the optimal subset of observed variables, necessitating iterative refinement. This typically involves running multiple EFA models to exclude less relevant variables or reduce the number of factors, aiming to achieve a robust and replicable factor structure that fits the data well. Based on the final EFA model, analysts are often encouraged to conduct a confirmatory factor analysis (CFA) to cross-validate the final factor structure under more restrictive conditions (Byrne, 1989).

Although variable selection algorithms (e.g., stepwise procedures, random forests) are widely used in statistics to refine predictive models and assess their stability across subsamples, few such algorithms have been developed specifically for factor analysis. In practice, variable selection in factor analysis remains relatively uncommon. Yet, these algorithms have significant potential to streamline the identification of the most relevant observed variables for a given factor structure and explore factor structure replicability. By reducing reliance on manual, iterative adjustments, they can enhance the generalizability, reproducibility, interpretability, and statistical robustness of factor analytic results. Such algorithms could also systematically evaluate variables based on their contributions to the underlying factors, helping analysts produce replicable factor structures across samples while minimizing subjectivity and human error. In addition, automating the evaluation of alternative models offers substantial efficiency gains by saving time and resources and providing a data-driven foundation for retention or removal decisions, ultimately improving the quality and consistency of factor analysis outcomes.

While manually removing observed variables and factors may seem appealing, allowing analysts to integrate their expertise and consider all available model information, this approach carries significant limitations that may undermine its credibility and replicability. Unlike established variable selection procedures, manual methods lack clearly defined, standardized rules and criteria, making them difficult to reproduce across analysts. In published research, these steps are often vaguely described, inconsistently applied, and heavily influenced by individual training and subjective judgment.

Perhaps most critically, manual approaches give little attention to the replicability of observed variables and factors. Without this evaluation, researchers remain unaware of whether different data or an alternative selection strategy would yield the same factor structure. As demonstrated in other areas of variable selection, resampling methods are indispensable for assessing and quantifying model stability (De Bin et al., 2016; Sauerbrei & Schumacher, 1992). Incorporating such methods into factor analysis not only aligns with best practices in statistical modeling but also provides a robust, data-driven foundation for producing reliable, generalizable, and replicable factor solutions.

Heinze et al. (2018) stated that effective algorithms should indicate (a) the frequency that an independent variable is selected, (b) sampling distributions of model coefficients, (c) the frequency of certain sets of independent variables, and (d) whether pairs of independent variables are competing for model selection. Building on these principles, the *Replicable Factor Analytic Solutions* (RFAS; Sass & Sanchez, 2023) algorithm was developed to meet these criteria and provide analysts with a factor analysis algorithm that could determine whether different factor structures are replicable across samples based on the analyst’s criteria (e.g., desired model complexity and level of model fit). Moreover, this algorithm provides evidence associated with the replicability of the set of observed variables and factors across both EFA and CFA models.

While the RFAS algorithm produces a recommended factor model, its output should serve as a decision-support tool rather than a prescriptive solution. Integrating the algorithm’s findings with theoretical considerations enables analysts to determine the most reproducible factor structure and evaluate whether additional variables should be retained based on both their replicability and theoretical relevance.

Several alternative approaches to variable selection in factor analysis have been proposed, including stochastic search factor selection (Mavridis & Ntzoufras, 2014), stepwise selection (Kano & Harada, 2000), Weighted Group Least Absolute Shrinkage and Selection Operator (LASSO) factor analysis (Hirose & Konishi, 2012), and Ant Colony Optimization (Marcoulides & Drezner, 2003). While each has notable strengths, these methods often fall short of the criteria outlined by Heinze et al. (2018) and frequently underperform when tasked with selecting models that exhibit strong factor structure properties. A primary limitation is their predominant focus on optimizing model fit, rather than adopting a holistic approach that also considers replicability and factor structure interpretability.

For example, the stepwise variable selection method has been shown to perform poorly (Kano & Harada, 2000) and, in some cases, even worse than the traditional practice of removing observed variables with loadings below 0.30 (Hogarty et al., 2004). This is not surprising given its common focus on model fit. Although the weighted group LASSO factor analysis approach has produced promising results in simulation studies, it does not account for model fit or replicability, and is unlikely to perform well when all variables exhibit at least one large factor loading. Ant Colony Optimization (ACO) offers a flexible metaheuristic search strategy but is sensitive to parameter choices (e.g., model fit criteria, number of factors, and desired number of observed variables per factor) and does not inherently assess replicability without additional resampling procedures. More broadly, existing variable selection methods often neglect the evaluation of replicability, do not systematically compare EFA and CFA solutions across subsamples, and provide limited flexibility for analysts to determine the optimal level of factor structure complexity.

In summary, a robust factor analysis algorithm would provide analysts with an objective alternative to factor structures derived solely from subjective judgment or theoretical assumptions. This parallels common practice in regression or classification modeling, where a theory-driven model is often supplemented with a variable selection algorithm to explore competing specifications. In factor analysis, the absence of such objective tools leaves analysts vulnerable to confirmation bias, believing that a particular structure exists despite limited empirical support, or to the application of suboptimal analytic practices (Schmitt et al., 2018).

## RFAS Description

The RFAS algorithm uses resampling methods to propose potential EFA and CFA solutions, while at the same time indicating what observed variables and factors were most often recovered (i.e., retained in a final factor solution based on a given algorithm criteria). The RFAS starts by creating R subsamples (i.e., R sets of training and validation subsamples). For each training subsample, the variable selection algorithm called Fit and Simple (FS) is applied, where observed variables are removed until a good-fitting approximate simple structure (or more complex if desired) is obtained (see Figure 1). The algorithm removes observed variables either sequentially (one observed variable at a time) or in tandem (all observed variables that do not meet the model criteria) based on a set of algorithm rules designed to eliminate observed variables deemed unimportant (i.e., do not load on any factors), complex (i.e., load on multiple factors), and/or display poor EFA model fit.

**Figure 1.**
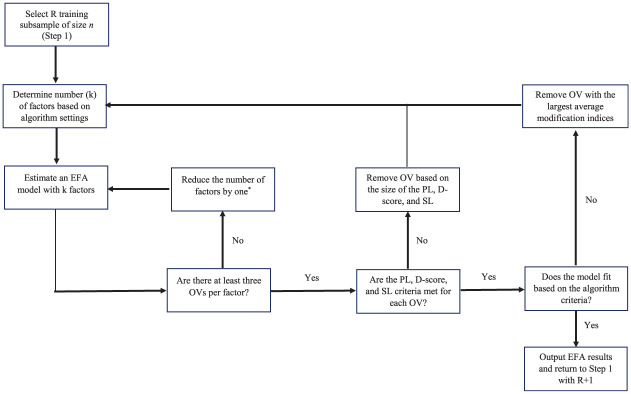
This diagram illustrates the iterative procedure used to identify replicable exploratory factor analytic solutions across training subsamples. Note. *R* = Training subsample, *n* = training subsample sample size, k = Number of factors, OV = Observed variable, PL = Primary factor loading, and SL = Secondary factor loading. *If there is only one factor the algorithm stops and outputs the results.

The FS algorithm is designed to identify not only the observed variables most likely to be retained in a final EFA solution but also the number of factors most consistently supported by the data. By systematically evaluating thousands of candidate models, the algorithm estimates the factor count most frequently reproduced (e.g., 80% of solutions yield three factors, 15% yield two factors, and 5% yield four factors) and calculates the proportion of times each observed variable is retained. This output provides critical evidence regarding which variables are most likely to appear in future analyses, thereby enhancing the replicability and stability of factor solutions. Although the algorithm generates a recommended model, it is intended as a decision-support tool rather than a prescriptive outcome. Researchers may, for instance, choose to retain a variable with a moderate replicability rate (e.g., retained in 65% of subsamples) to preserve content validity, while acknowledging that its exclusion in other runs resulted from issues such as model misfit (20%), low factor loadings (15%), or cross-loadings (10%).^1^

Building on the final model results from the FS algorithm, the second stage of the RFAS procedure, the Replicable Fit and Simple (RFS) algorithm, uses the R validation subsamples to re-estimate the factor model within a CFA framework. This step evaluates CFA model quality, including global fit indices, factor loadings, and interfactor correlations, under more restrictive conditions in which all cross-loadings are fixed to zero and error variances are assumed to be independent. In other words, the RFS algorithm assesses the reproducibility and quality of the CFA model implied by the factor structure identified in the FS stage.

Although a detailed discussion of the RFAS algorithm is provided by Sass and Sanchez (2023), a brief overview of the configurable settings is presented here. Prior to running the algorithm, the analyst must specify the following parameters: (a) the number of subsamples used to examine the data (default: *R* = 100); (b) the proportion of the full sample allocated to the training set (default: 66%) and validation set (default: 34%); (c) the minimum magnitude of an observed variable’s primary factor loading required for retention (default: λ >|0.40|); (d) the maximum magnitude of an observed variable’s secondary factor loadings permitted for retention (default: λ <|0.30|); (e) the D-score threshold for variable retention (default: *D* = 3; see Sass & Sanchez, 2023 for details); (f) the minimum proportion of subsample EFA solutions in which an observed variable must appear to be included in the final FS model (default: 80%); (g) the estimation method (default: weighted least squares with mean and variance adjustment [WLSMV]); (h) the rotation method (default: Geomin); (i) the target number of factors or the method used to determine it (default: parallel analysis); (j) the model fit criteria (default: CFI and TLI > .90, SRMR < 0.10); and (k) the removal strategy for observed variables (default: variables removed sequentially, meaning one at a time).

## Study Purpose

The present study was designed to rigorously evaluate the performance and utility of the RFAS algorithm for factor analytic practice, with particular focus on its robustness, flexibility, and replicability across diverse analytic conditions. Study 1 systematically assessed the algorithm across a wide range of simulated factor structures, examining how modifications to default parameter settings influence the reproducibility and quality of the resulting solutions. This evaluation also tested the algorithm’s ability to produce replicable factor structures under varying sample sizes, levels of factor complexity, and magnitudes of interfactor correlations, thereby identifying conditions under which the algorithm performs optimally and where it may fail under default settings. Study 2 extended this assessment by benchmarking RFAS against established variable selection methods, including ACO, weighted group LASSO, and stepwise selection methods, to determine its relative strengths and limitations. Together, these studies provide a comprehensive evaluation of RFAS, offering practical guidance for researchers seeking to apply factor analysis algorithms in empirical research.

## Methods

### Study 1: Evaluation of RFAS

#### Simulated Data

A simulation study was designed to test the effects of different factor structures on the RFAS’s performance. Six models (see Table 1) with 54 total experimental conditions (6 factor models, 3 sample sizes, and 3 interfactor correlation magnitudes) were selected to consider common factor structures found in empirical and simulation studies. More specifically, the 54 factor analytic conditions were analyzed to explore six-factor structures (four closer to an approximate simple structure and two more complex structures), three sample sizes (*n* = 300, *n* = 500, *n* = 1000), and three interfactor correlation magnitudes (ρ = 0, ρ =.3, ρ =.6).

**Table 1.** Summary of Six Factor Models Explored

Model	NF	NV	PLPF	Structure	MP	MS	IFC	*n*
Model 1	4	40	10, 10, 10, 10	AS	0.56	0.08	0, .3, .6	300, 500, 1000
Model 2	4	40	10, 10, 10, 10	AS	0.55	0.12	0, .3, .6	300, 500, 1000
Model 3	4	26	17,5,2,2	AS	0.63	0.09	0, .3, .6	300, 500, 1000
Model 4	4	20	5,5,5,5	AS	0.58	0.14	0, .3, .6	300, 500, 1000
Model 5	4	40	10, 10, 10, 10	C	0.62	0.15	0, .3, .6	300, 500, 1000
Model 6	4	26	14,4,4,4	C	0.60	0.15	0, .3, .6	300, 500, 1000

Note. NF = Number of Factors, NV = Number of Variables, PLPF = Primary Loadings per Factor, AS = Approximately Simple structure, C = Complex structure, MP = mean primary loading, MS = mean secondary loading, IFC = Interfactor Correlation, *n* = Sample Size.

To create these data,^2^ multivariate normal continuous random observed variables were generated within Mplus 8.2 based on the models in Table 1. Each observed variable was independent and identically distributed, with the residuals being calculated as follows:

$$\psi =1-\sum_{k=1}^{m}\lambda_{k}^{2}+\sum_{j\neq k}\lambda_{j}\lambda_{k}r_{\mathrm{jk}},$$

where $\lambda_{k}$ is the factor loading on the *k*^th^ factor and $r_{\mathrm{jk}}$ is the interfactor correlation. The factor structures selected were chosen to present cases when questionable final factor solutions might be obtained (e.g., smaller sample size, highly correlated factors, and/or complex factor solutions) and cases when an acceptable final factor structure should be obtained (e.g., larger sample size, less correlated factors, and approximate simple structure solutions) with algorithm defaults.

Two important considerations should be noted in the evaluation of the model. First, because the sample data were drawn from the population, sampling error is present. Second, the simulated population parameters and those obtained through Geomin rotation (i.e., ρ and λ) exhibited slight discrepancies, as would be expected with any rotated solution. Given the large number of factor structures examined (54 simulated, 54 Geomin rotated, and 54 sample-based), a summary of these results is provided below, with full details to be made available online following acceptance of the manuscript.

#### Experimental Conditions

##### Sample Size

Past research (Hogarty et al., 2005; MacCallum et al., 1999) has thoroughly evaluated the minimum sample size requirement for stable factor loading estimates. Literature reviews have also indicated that most published research using EFA utilized sample sizes of roughly 300 (Fabrigar et al., 1999; Ford et al., 1986; Russell, 2002; Sass & Schmitt, 2010). Sample sizes of 100, 200, 300, and 500 are considered “poor,”“fair,”“good,”“very good,” and “excellent,” respectively (Comrey & Lee, 1992, pp. 216–217). To ensure stable factor loading estimates and obtain adequate split samples, sample sizes used in this study were 300 (*n*_training_ = 198 and *n*_validation_ = 102), 500 (*n*_training_ = 330 and *n*_validation_ = 170), and 1,000 (*n*_training_ = 660 and *n*_validation_ = 340). Note that these training and validation sample sizes only relate to the default setting (i.e., 66% of data used for training and 34% for validation). This study elected not to explore sample sizes less than 300, given that the sample sizes would arguably be too small for the training and validation data. Researchers with smaller sample sizes would likely benefit from using only the FS portion of the algorithm, rather than conducting cross-validation with the CFA models.

##### Correlation Between Factors and Rotation Criteria

To assess the impact of the interfactor correlation magnitude, three population simulation values were selected: 0, 0.3, and 0.6. These interfactor correlations should represent the range of correlations commonly encountered in practice, without the concern of multicollinearity (ρ’s > .|70|) between factors. Again, it is important to reiterate that the sample correlations differed due to sampling error and the rotation method selected.

##### Factor Structures

Two literature review studies (Conway & Huffcutt, 2003; Henson & Roberts, 2006) summarized previous empirical EFA studies in social and behavioral research. Henson and Roberts (2006) indicated the median number of extracted factors was three (ranging from one to seven) and the median number of observed variables was 20 (ranging from 5 to 110). Given this information and considering that the RFAS algorithm was designed to remove observed variables and factors as needed, the six primary factor structures contained more factors and observed variables than typically encountered in practice. These factor models are summarized below, with the actual sample and population factor structures being provided online after the paper is accepted for publication.

##### Model 1

This factor model (see Table 1, Model 1) with 40 observed variables represents a nearly perfect simple structure (i.e., large primary factor loadings and very small secondary loadings) with four factors and ten observed variables per factor. Based on this structure, the RFAS should remove relatively few observed variables and display a stable and replicable final solution using the default settings. However, the results are likely to be less replicable with smaller sample sizes (*n* = 300), especially with more highly correlated factors. Model 1 was designed to demonstrate the RFAS’s performance with clean factor structures.

##### Model 2

This factor model (see Table 1, Model 2) represents an approximate simple structure that mimics Model 1 (i.e., four factors and ten observed variables per factor), but with several larger secondary loadings. Hence, the RFAS algorithm should remove a larger number of observed variables due to these larger secondary loadings, while still displaying relatively stable and replicable final solutions. The number of factors is expected to typically remain at four because of the large number (*i* = 10) of initial observed variables per factor, with the algorithm selecting only those observed variables that are more replicable. Once again, the final model results are likely to be less replicable with smaller sample sizes (*n* = 300) and more highly correlated (ρ = .60) factors. Model 2 was designed to illustrate the RFAS’s ability to identify the best subset of replicable observed variables and factors under conditions when certain observed variables likely need to be removed to obtain a replicable simple factor structure.

##### Model 3

For this factor model (see Table 1, Model 3), a single strong factor was generated (i.e., factor 1 had 17 observed variables with larger primary loadings) and three weaker factors. Factor 2 had five observed variables, whereas factors 3 and 4 only had two observed variables per factor. This factor structure was generated to evaluate how the RFAS algorithm performs when the first factor is well defined, but the remaining factors are questionable. Specifically, Factor 2 may lack replicability due to the limited number of observed variables, whereas Factors 3 and 4 are likely spurious and should be removed.

##### Model 4

This model (see Table 1, Model 4) was generated to examine the RFAS algorithm’s performance with a smaller number of observed variables (*i* = 5) per factor (*f* = 4) under an approximate simple structure solution. While the algorithm is hypothesized to produce relatively replicable solutions with larger sample sizes (*n* = 1000) and smaller interfactor correlations (e.g., ρ≤ .3), the results are less certain with smaller sample sizes (*n*≤ 500) and/or more highly correlated factors (ρ = .60). The algorithm requires a minimum of three observed variables per factor, so the factor structure replicable may be less under default settings.

##### Model 5

This four-factor model with 10 observed variables per factor represents a complex structure (see Table 1, Model 5). The RFAS algorithm should remove a larger number of observed variables due to the larger secondary loadings, yet still display a replicable final solution, assuming enough (i.e., *i*≥ 3) observed variables remain per factor. The number of factors is expected to remain at four because of the large number (*i* = 10) of initial observed variables per factor; however, the number of observed variables per factor should be significantly smaller due to the initial complex structure and may be heavily dependent on the sample size (e.g., when *n* = 300) and intercorrelation (e.g., when ρ = .6).

##### Model 6

This model (see Table 1, Model 6) was designed to examine the algorithm’s performance under a more difficult situation, meaning a more complex factor structure and a smaller number of observed variables (i.e., four) on three of the four factors. The dominant factor had 14 observed variables. This factor structure configuration was chosen to assess whether the algorithm, when applied with its default settings, could recover a usable and replicable solution under more challenging data conditions, especially with smaller sample sizes and highly correlated factors.

#### RFAS Algorithm Defaults

The RFAS algorithm defaults used in this study were as follows: 100 subsamples for cross-validation (66% and 34% of the sample were used for the EFA training sets and CFA validation sets, respectively), primary loadings ≥|0.40,| secondary loadings ≤|0.30,| D-score > 3, CFI and TLI ≥ 0.90, SRMR and RMSEA ≤ 0.10, replicability rating (i.e., proportion of times an observed variable is retains in the final solution) of 80%, the sequential method with maximum likelihood estimation, and oblique Geomin factor rotation.

For comparative purposes, both parallel analysis (PA) and Exploratory Graph Analysis (EGA) were employed to identify the appropriate number of factors. While PA is a more conventional approach, empirical evidence suggests that EGA demonstrates superior performance with highly correlated factors (Golino et al., 2020; Golino & Epskamp, 2017). Following the identification of a final EFA solution, defined as a subset of observed variables achieving at least an 80% replicability threshold, the same training and validation subsamples were subsequently employed to evaluate the final EFA and CFA models’ replicability.

Although not done here, if the final model were deemed unsatisfactory (e.g., due to the elimination of a substantial number of observed variables or usable factors), the algorithm settings could be modified and the model re-estimated under less restrictive conditions (see Sass & Sanchez, 2023). Even when an acceptable solution is achieved using the default settings, conducting sensitivity analyses by making slight adjustments to the algorithm parameters is advisable. In addition, researchers may use the information generated (e.g., replicability ratings, the number of factors most consistently retained, and the rationale for the exclusion of specific observed variables) to construct a theoretically informed factor model that integrates both substantive content considerations and guidance from the FS algorithm.

#### Modified RFAS Algorithm Settings

The results indicated that several models demonstrated poor outcomes, particularly with respect to observed variable or factor replicability. In these instances, modified algorithm settings were applied to reexamine the “questionable” factor structures, drawing on the Reasons table (i.e., it indicates the reason each observed variable was removed) as well as the replicability ratings of the observed variables (see *Modified Algorithm Results*). Note, a questionable algorithm outcome should not be interpreted as evidence of algorithmic failure; rather, it signifies a factor structure that lacks replicability based on those algorithm settings (e.g., an approximate simple structure).

### Study 2: Comparative Performance Across Variable Selection Methods

The purpose of the second study was to compare the performance of four variable selection methods for factor analysis: RFAS, ACO, Weighted Group LASSO (WGL), and two Stepwise approaches (one using a TLI target criterion and the other using a ΔTLI criterion). Rather than replicating the full set of 108 experimental conditions from Study 1, Study 2 examined a subset of ten diverse factor structures selected for comparison purposes. These structures were intentionally chosen to reflect a range of model complexities (pure, approximate, and complex factor structures), sample sizes, numbers of variables per factor, and inter-factor correlations commonly encountered in applied research.

As shown in Table 2, the conditions generally involved moderate to large inter-factor correlations (five conditions with *r* = .30 or .60, and none with *r* = 0) and sample sizes typically considered adequate for stable parameter estimation in factor analysis (*n* = 500 or 1000; see Fabrigar et al., 1999; MacCallum et al., 1999). An exception was the second condition, which employed a simple factor structure with a smaller sample (*n* = 300) to evaluate algorithm performance under more constrained conditions. Conditions with *r* = 0 were intentionally excluded, as such cases rarely occur in empirical research. By incorporating both favorable and constrained scenarios, the study aimed to evaluate the robustness, parsimony, and stability of the algorithms across a range of plausible research contexts.

**Table 2.** Model Fit Results for the Ten-Factor Models Explored in Study 2

Model (IFC, *n*, OV)	CFA			EFA		
	CFI	TLI	RMSEA	CFI	TLI	RMSEA
Model 1 (0.3, 1000, 40)	0.986	0.985	0.017	1.000	1.002	0.000
Model 1 (0.6, 300, 40)	0.993	0.993	0.015	0.997	0.996	0.011
Model 2 (0.3, 500, 40)	0.846	0.836	0.058	1.000	1.002	0.000
Model 2 (0.6, 1000, 40)	0.921	0.916	0.050	1.000	1.002	0.000
Model 3 (0.6, 1000, 26)	0.937	0.929	0.055	0.995	0.994	0.160
Model 4 (0.3, 1000, 20)	0.888	0.871	0.076	0.999	0.998	0.009
Model 5 (0.6, 500, 40)	0.928	0.924	0.066	0.999	0.999	0.009
Model 5 (0.3, 1000, 40)	0.878	0.871	0.068	1.000	1.001	0.000
Model 6 (0.3, 500, 26)	0.893	0.881	0.070	0.998	0.998	0.010
Model 6 (0.6, 500, 26)	0.948	0.943	0.061	0.999	0.999	0.009

Note. The interfactor correlation (IFC), number of observations (*n*), and number of variables (OV) per condition in each model.

#### Algorithm Comparison

This study compared the performance of five variable selection methods: RFAS, ACO, Weighted Group LASSO (WGL), and two Stepwise procedures (one based on a TLI target criterion and the other based on a ΔTLI criterion). Descriptions of the RFAS and ACO methods are provided in the preceding and subsequent sections, respectively, while the remaining three methods are documented in greater detail online, along with the corresponding code and results, for readers interested in implementation (see RFAS website).

For the WGL method, this study extended the approach of Hirose and Konishi (2012) by implementing 100 subsamples, each based on 66% of the data, and computing the Jaccard similarity coefficient to assess stability. The frequency with which each observed variable was retained across replications was recorded. Because variables were rarely removed, detailed results are not reported and are instead available online. This outcome was primarily attributable to the factor structures examined, where most observed variables exhibited strong, large primary loadings on at least one factor, making it unlikely that the entire row of loadings (i.e., observed variable) would be reduced to zero.

The stepwise methods (Kano & Harada, 2000) were applied using the same subsampling framework (100 replications with 66% of the data) and identical evaluation metrics. Similar to the WGL method, these procedures rarely eliminated observed variables. This outcome could have been anticipated, given that the EFA structures under investigation already exhibited satisfactory model fit indices (see Table 2), indicating that further variable elimination to improve the model fit was unnecessary to obtain acceptable solutions.

Taken together, the outcomes for WGL and the Stepwise approaches highlight an important contextual boundary for variable selection methods. Specifically, when factor structures already exhibit strong primary loadings and adequate global fit, algorithms designed to eliminate poorly performing variables based on these criteria are unlikely to make substantial modifications. That said, RFAS and ACO are designed to probe to identify smaller subsets of variables even when the EFA model fits the data and primary loadings are large.

#### Ant Colony Optimization (ACO) Algorithm for Factor Analysis

ACO is a metaheuristic inspired by the foraging behavior of ants and was originally developed to address complex combinatorial optimization problems (Dorigo, 1992). In psychometrics, it has been adapted for observed variable selection, particularly in short form development and measurement refinement (Leite et al., 2008; Marcoulides & Drezner, 2003). Building on this prior work, the present study employed ACO to identify subsets of observed variables that optimized model fit within a CFA framework.

In the context of this study, ACO was employed to identify subsets of observed variables that optimize model fit within a CFA framework. The algorithm begins by initializing pheromone levels across all observed variables. Artificial “ants” then construct candidate subsets by probabilistically selecting observed variables, balancing pheromone strength (indicating prior success) with heuristic information, such as factor loadings. Each subset is evaluated using CFA, with model fit assessed via multiple indices. Following evaluation, pheromone levels are updated to reinforce high-performing observed variable selections. This process iterates until convergence or until a predetermined number of iterations is reached.

ACO’s capacity to simultaneously optimize multiple, and sometimes competing, criteria gives it an advantage over traditional heuristic methods that rely solely on simple metrics (e.g., highest loading or item-total correlation). Its flexibility has been demonstrated in various applications, including scale abbreviation (Leite et al., 2008), measurement invariance (Jankowsky et al., 2020), model specification within structural equation modeling (Marcoulides & Drezner, 2003), and measurement development (Dong & Dumas, 2025).

#### ACO Model Estimation

The CFA short-form selection procedure was implemented in R using the *lavaan* package for CFA model estimation and *ShortForm* for ACO search. The targeted observed variables per factor size varied across conditions (see Table 3) to mimic what applied research might use in practice and to maintain a minimum of three items per factor for statistical identifiability. Note that changing these algorithm settings could significantly alter the model results.

**Table 3.** Provide the Actual and Targeted Variables Per Factor and the Average Jaccard Statistic for the ACO Method

Model (IFC, *n*, OV)	a.per.f	v.per.f	Average Jaccard statistic
Model 1 (0.3, 1000, 40)	10,10,10,10	7,7,7,7	0.618
Model 1 (0.6, 300, 40)	10,10,10,10	7,7,7,7	0.619
Model 2 (0.3, 500, 40)	10,10,10,10	5,5,5,5	0.430
Model 2 (0.6, 1000, 40)	10,10,10,10	5,5,5,5	0.445
Model 3 (0.6, 1000, 26)	17,5,2,2	10,5	0.539
Model 4 (0.3, 1000, 20)	5,5,5,5	4,4,4,4	0.835
Model 5 (0.6, 500, 40)	10,10,10,10	5,5,5,5	0.408
Model 5 (0.3, 1000, 40)	10,10,10,10	5,5,5,5	0.436
Model 6 (0.3, 500, 26)	14,4,4,4	10,3,3,3	0.694
Model 6 (0.6, 500, 26)	14,4,4,4	10,3,3,3	0.710

Note. a.per.f = actual number of variables per factor in the data, whereas v.per.f = the targeted number of variables per factor.

The ACO search was configured to identify observed variables subsets that optimized model fit while satisfying theoretical and statistical constraints. The primary search used 15 artificial ants per iteration to balance solution space exploration with computational efficiency. The pheromone evaporation rate was set to 0.85 to retain useful search information while preventing early convergence to suboptimal solutions. Each ant performed 20 construction steps per run, and the algorithm was allowed up to 150 runs to increase the likelihood of obtaining high-quality solutions. Candidate models were evaluated using the CFI, TLI, and RMSEA, with an initial screening criterion of CFI ≥ .90, TLI ≥ .90, and RMSEA ≤ .08, consistent with recommended “adequacy” thresholds set by Hu and Bentler (1999), and to ensure a subset of observed variables produced an acceptable solution. Model estimation used ML with robust (MLR) standard errors given the continuous nature of the data.

One significant limitation of using ACO for variable selection within the *ShortForm* package is that users must set the number of observed variables per factor. Consequently, meaningful observed variables could be omitted from the final model, and less meaningful ones could be included, assuming the algorithm criteria are met. Therefore, following completion of the global ACO search, which identifies the best-performing short-form model from the entire combinatorial search space, the algorithm entered a *local neighborhood search* phase.

This *local neighborhood search* process refinement systematically examined small, targeted modifications to the ACO-best model to determine whether incremental observed variable substitutions could further improve model fit. The local neighborhood was defined by single-observed variable swaps within each factor, meaning that, for a given factor, one existing observed variable in the short-form solution could be removed and replaced with an unused observed variable from that factor’s complete observed variable pool. This procedure was constrained by allowing at most three observed variables to be candidates for removal and at most five unused observed variables to be candidates for addition per factor. These limits were set to balance computational efficiency with adequate exploration of plausible alternative configurations, thereby preventing an exhaustive but computationally prohibitive search.

For each candidate swap, the revised factor–observed variable configuration was estimated with a CFA model using the full dataset. The retained neighbor models were then evaluated on the same global fit indices (CFI, TLI, & RMSEA) and compared against the original ACO-best model to determine whether any local swaps yielded superior fit without sacrificing model parsimony or theoretical coherence. This step ensured that the final selected model represented not just a global optimum from the ACO search, but also a locally optimized configuration resistant to minor perturbations.

To assess the stability and reproducibility of each model, 100 subsamples were drawn without replacement, with each subsample consisting of 66% of the original observations. The ACO procedure described above was then applied to each subsample. Pairwise Jaccard similarity coefficients were computed across the 100 resulting solutions, and the mean coefficient (see Table 3) was used as an index of variable selection consistency. In addition, the algorithm reported the proportion of replications in which each observed variable was retained in the final model.

## Results

### Study 1. Default Algorithm Results

#### Model 1

The results from Model 1 performed well as expected, given the approximate simple factor structure and large number of observed variables (i.e., ten) per factor. Model 1 summary results (see Table 4) revealed that the algorithm identified a four-factor solution with high replicability ratings and a large percentage of observed variables across most conditions. The exception was for highly correlated factors^3^ using parallel analysis, which resulted in the algorithm having a low replicability rating (e.g., 53% for *n* = 500 & 68% for *n* = 1000), difficulty distinguishing between factors (i.e., less than three factors per condition), and a poor fitting EFA and CFA model for the *n* = 300 and *n* = 500 conditions. Conversely, EGA identified a four-factor solution with highly correlated factors and produced good fitting models across all nine conditions. This finding is potentially significant, as it suggests that EGA works better with more highly correlated factors. Another noteworthy, yet expected, finding is the percentage of observed variables retained tended to increase with larger sample sizes.

**Table 4.** Summary of Final Factor Analysis Across Conditions for Model 1

Model	IFC	*n*	NF (P,A,F)	Replicability	OV (P,S,%)	EFA model fit	CFA model fit
*Parallel Analysis*							
Model 1	0	300	4,4,4	100%	40,34,85	Yes	Yes
Model 1	0	500	4,4,4	100%	40,36,90	Yes	Yes
Model 1	0	1000	4,4,4	100%	40,36,90	Yes	Yes
Model 1	.3	300	4,4,4	100%	40,34,85	Yes	Yes
Model 1	.3	500	4,4,4	100%	40,37,92	Yes	Yes
Model 1	.3	1000	4,4,4	100%	40,37,92	Yes	Yes
Model 1	.6	300	4,1,1	100%	40,33,82	No	No
Model 1	.6	500	4,1,1	53%	40,32,80	No	No
Model 1	.6	1000	4,2,2	68%	40,30,75	Yes	Yes
*Exploratory Graph Analysis*							
Model 1	0	300	4,4,4	100%	40,34,85	Yes	Yes
Model 1	0	500	4,4,4	100%	40,36,90	Yes	Yes
Model 1	0	1000	4,4,4	100%	40,36,90	Yes	Yes
Model 1	.3	300	4,4,4	100%	40,34,85	Yes	Yes
Model 1	.3	500	4,4,4	100%	40,37,92	Yes	Yes
Model 1	.3	1000	4,4,4	100%	40,37,92	Yes	Yes
Model 1	.6	300	4,4,4	97%	40,31,78	Yes	Yes
Model 1	.6	500	4,4,4	100%	40,35,88	Yes	Yes
Model 1	.6	1000	4,4,4	100%	40,37,92	Yes	Yes

Note. IFC = Interfactor correlations; *n* = sample size; NF = Number of factors in population (P), number of factors after the algorithm (A), and the number of factors in the final model (F); Replicability = Percent of subsamples replicated with that number of factors; OV = Number of observed variables in population (P), number of observed variables in the sample (S) after the FS algorithm, and percent (%) of observed variables after the FS algorithm; Model fit = Whether the final model fit the data across the four fit statistics (i.e., CFI & TLI > .95 and RMSEA and SRMR < .08).

#### Model 2

The simulated data for Model 2 mimicked Model 1, except larger secondary factor loadings (i.e., increased factor structure complexity). Similar to Model 1 results, as the interfactor correlation increased, the number of retained factors and observed variables tended to decrease (see Table 5). These results might be expected, given that increased model complexity should result in the removal of more observed variables, and factor analysis tends to struggle to distinguish between highly correlated factors. For this model, parallel analysis and EGA produced broadly comparable results, with neither method able to retain four factors when using the default algorithm settings. However, parallel analysis generally demonstrated lower replicability than EGA, particularly under the *n* = 300 and *n* = 500 conditions.

**Table 5.** Summary of Final Factor Analysis Across Conditions for Model 2

Model	IFC	*n*	NF (P,A,F)	Replicability	OV (P,S,%)	EFA model fit	CFA model fit
*Parallel Analysis*							
Model 2	0	300	4,4,4	97%	40,21,52	Yes	No
Model 2	0	500	4,4,4	99%	40,23,57	Yes	No
Model 2	0	1000	4,4,4	100%	40,28,70	Yes	No
Model 2	.3	300	4,4,3	60%	40,16,40	No	No
Model 2	.3	500	4,4,4	94%	40,24,60	Yes	No
Model 2	.3	1000	4,4,4	100%	40,27,68	Yes	No
Model 2	.6	300	4,2,1	47%	40,7,18	No	No
Model 2	.6	500	4,3,2	46%	40,12,30	Yes	Yes
Model 2	.6	1000	4,3,3	86%	40,18,45	Yes	Yes
*Exploratory Graph Analysis*							
Model 2	0	300	4,4,4	97%	40,21,52	Yes	No
Model 2	0	500	4,4,4	99%	40,23,57	Yes	No
Model 2	0	1000	4,4,4	100%	40,28,70	Yes	No
Model 2	.3	300	4,4,4	79%	40,16,40	Yes	No
Model 2	.3	500	4,4,4	94%	40,24,60	Yes	No
Model 2	.3	1000	4,4,4	100%	40,27,68	Yes	No
Model 2	.6	300	4,3,2	79%	40,9,22	Yes	Yes
Model 2	.6	500	4,3,2	78%	40,12,30	Yes	Yes
Model 2	.6	1000	4,3,3	73%	40,19,48	Yes	Yes

Note. IFC = Interfactor correlations; *n* = sample size; NF = Number of factors in population (P), number of factors after the algorithm (A), and the number of factors in the final model (F); Replicability = Percent of subsamples replicated with that number of factors; OV = Number of observed variables in population (P), number of observed variables in the sample (S) after the FS algorithm, and percent (%) of observed variables after the FS algorithm; Model fit = Whether the final model fit the data across the four fit statistics (i.e., CFI & TLI > .95 and RMSEA and SRMR < .08).

In general, the algorithm performed as expected. It removed observed variables that did not conform to a simple structure solution. Based on these results, the analyst could change the algorithm criteria to retain more observed variables (and potentially factors), but that might come at the cost of increased model complexity and a poorer CFA model fit. Of course, this might be a perfectly acceptable factor solution based on the analyst’s needs and goals.

#### Model 3

This model contained 17 and five observed variables on factors 1 and 2, respectively, and only two observed variables on factors 3 and 4. This information is important given that a two-factor solution should be expected, with at least 15% (4 of the 26) of the observed variables expected to be removed (see Table 6). In all cases, a two-factor solution was obtained with typically about 22 observed variables remaining in the final model. Only two conditions (*r* = .6, *n* = 300 for PA and EGA) lost more than 85% of the observed variables. The percentage of retained factors also remained relatively stable across both sample size and interfactor correlation conditions. Moreover, both the EFA and CFA models consistently demonstrated good model fit statistics.

**Table 6.** Summary of Final Factor Analysis Across Conditions for Model 3

Model	IFC	*n*	NF (P,A,F)	Replicability	OV (P,S,%)	EFA model fit	CFA model fit
*Parallel Analysis*							
Model 3	0	300	4,2,2	100%	26,22,85	Yes	No
Model 3	0	500	4,2,2	100%	26,22,85	Yes	No
Model 3	0	1000	4,2,2	100%	26,22,85	Yes	No
Model 3	.3	300	4,2,2	96%	26,22,85	Yes	No
Model 3	.3	500	4,2,2	100%	26,22,85	Yes	No
Model 3	.3	1000	4,2,2	100%	26,22,85	Yes	No
Model 3	.6	300	4,2,2	93%	26,20,77	Yes	Yes
Model 3	.6	500	4,2,2	100%	26,22,85	Yes	Yes
Model 3	.6	1000	4,2,2	100%	26,22,85	Yes	Yes
*Exploratory Graph Analysis*							
Model 3	0	300	4,2,2	100%	26,22,85	Yes	No
Model 3	0	500	4,2,2	100%	26,22,85	Yes	No
Model 3	0	1000	4,2,2	100%	26,22,85	Yes	No
Model 3	.3	300	4,2,2	96%	26,22,85	Yes	No
Model 3	.3	500	4,2,2	100%	26,22,85	Yes	No
Model 3	.3	1000	4,2,2	100%	26,22,85	Yes	No
Model 3	.6	300	4,2,2	83%	26,20,77	Yes	Yes
Model 3	.6	500	4,2,2	97%	26,22,85	Yes	Yes
Model 3	.6	1000	4,2,2	100%	26,22,85	Yes	Yes

Note. IFC = Interfactor correlations; *n* = sample size; NF = Number of factors in population (P), number of factors after the algorithm (A), and the number of factors in the final model (*F*); Replicability = Percent of subsamples replicated with that number of factors; OV = Number of observed variables in population (P), number of observed variables in the sample (S) after the FS algorithm, and percent (%) of observed variables after the FS algorithm; Model fit = Whether the final model fit the data across the four fit statistics (i.e., CFI & TLI > .95 and RMSEA and SRMR < .08).

#### Model 4

For this model, four factors (each with five observed variables) were generated with a rather complex factor structure. Given this factor structure’s complexity and the relatively small number of observed variables per factor, the factor replicability rating, percent of observed variables retained, and model fit statistics differed significantly as a function of both the interfactor correlation and sample size (see Table 7). In terms of the final number of factors, the algorithm never retained a four-factor solution. Parallel analysis and EGA produced the same number of factors for the final solution under the ρ = 0 and .3 interfactor correlation conditions (regardless of sample size), whereas EGA often (i.e., for the *n* = 300 and 500 conditions) retained more factors in the final solution under the ρ = .6 conditions. In all cases, the number of factors in the original data set (i.e., with all 20 observed variables) was one with parallel analysis and two with EGA.

**Table 7.** Summary of Final Factor Analysis Across Conditions for Model 4

Model	IFC	*n*	NF (P,A,F)	Replicability	OV (P,S,%)	EFA model fit	CFA model fit
*Parallel Analysis*							
Model 4	0	300	4,2,2	54%	20,6,30	Yes	Yes
Model 4	0	500	4,3,3	89%	20,9,45	Yes	No
Model 4	0	1000	4,3,3	82%	20,12,60	Yes	No
Model 4	.3	300	4,2,2	74%	20,7,35	Yes	Yes
Model 4	.3	500	4,3,3	79%	20,11,55	Yes	No
Model 4	.3	1000	4,3,3	84%	20,11,55	Yes	No
Model 4	.6	300	4,1,1	100%	20,11,55	No	No
Model 4	.6	500	4,1,1	99%	20,10,50	No	No
Model 4	.6	1000	4,1,1	95%	20,14,70	No	No
*Exploratory Graph Analysis*							
Model 4	0	300	4,2,2	51%	20,6,30	Yes	Yes
Model 4	0	500	4,3,3	89%	20,9,45	Yes	No
Model 4	0	1000	4,3,3	82%	20,12,60	Yes	No
Model 4	.3	300	4,2,2	70%	20,6,30	Yes	Yes
Model 4	.3	500	4,3,3	81%	20,11,55	Yes	No
Model 4	.3	1000	4,3,3	86%	20,11,55	Yes	No
Model 4	.6	300	4,2,2	92%	20,6,30	Yes	No
Model 4	.6	500	4,2,2	82%	20,10,50	Yes	Yes
Model 4	.6	1000	4,2,1	50%	20,6,30	No	No

Note. IFC = Interfactor correlations; *n* = sample size; NF = Number of factors in population (P), number of factors after the algorithm (A), and the number of factors in the final model (F); Replicability = Percent of subsamples replicated with that number of factors; OV = Number of observed variables in population (P), number of observed variables in the sample (S) after the FS algorithm, and percent (%) of observed variables after the FS algorithm; Model fit = Whether the final model fit the data across the four fit statistics (i.e., CFI & TLI > .95 and RMSEA and SRMR < .08).

Although EGA retains more factors under the ρ = .6 conditions, the replicability rating was lower for EGA compared to parallel analysis. In addition, a larger percentage of the observed variables was removed with EGA under the ρ = .6 with *n* = 300 and 1000 conditions. Notice that the greater model complexity is also evident, given that the CFA models often did not fit the data well. Overall, these findings suggest that a replicable pure simple structure is difficult to retain under such factor structure conditions, thus likely generating vastly different factor solutions across research studies and researchers.

To create a more replicable solution, the analyst would likely have to change the RFAS algorithm settings to allow more model complexity and perhaps reduce the required observed variable’s replicability rating. These findings would also suggest to researchers that scale/measurement revisions might be required to obtain consistent results across studies. Users should make use of the “Reasons” table that indicates why each observed variable was removed to make the algorithm modifications, as this should provide insight related to how the scale is revised (e.g., do observed variables not consistently load strongly on a factor, do they often cross-load, or is there a problem related to misfit).

#### Model 5

For this model, a complex factor structure was simulated to have four factors and ten observed variables per factor. As might be expected due to the increased model complexity, the RFAS algorithm often did not retain four factors, and a large percentage of observed variables were often removed (see Table 8). Relatedly, the factor replicability ratings varied considerably (i.e., from 52% to 95%), thus implying that the factor structure generated varied significantly as a function of the sample size and interfactor correlation. In fact, more than half of the observed variables were removed in all final factor solutions, except for when *n* = 300, ρ = .6, and parallel analysis was used to select the number of factors. Overall, this model provides an excellent example of when the algorithm settings should be revised to allow for greater model complexity, or the analyst would need to revise/add observed variables that promote a simpler structure (if that is the desired outcome).

**Table 8.** Summary of Final Factor Analysis Across Conditions for Model 5

Model	IFC	*n*	NF (P,A,F)	Replicability	OV (P,S,%)	EFA model fit	CFA model fit
*Parallel Analysis*							
Model 5	0	300	4,3,2	74%	40,14,35	Yes	No
Model 5	0	500	4,3,2	75%	40,9,22	Yes	No
Model 5	0	1000	4,3,2	88%	40,9,22	Yes	No
Model 5	.3	300	4,2,2	60%	40,9,22	Yes	Yes
Model 5	.3	500	4,3,3	85%	40,12,30	Yes	Yes
Model 5	.3	1000	4,3,3	95%	40,15,38	Yes	No
Model 5	.6	300	4,2,2	79%	40,23,57	No	No
Model 5	.6	500	4,2,2	81%	40,14,35	Yes	Yes
Model 5	.6	1000	4,2,2	64%	40,15,38	Yes	Yes
*Exploratory Graph Analysis*							
Model 5	0	300	4,3,2	73%	40,13,32	Yes	No
Model 5	0	500	4,3,2	73%	40,9,22	Yes	No
Model 5	0	1000	4,3,2	88%	40,9,22	Yes	No
Model 5	.3	300	4,3,2	69%	40,12,30	Yes	Yes
Model 5	.3	500	4,3,3	82%	40,12,30	Yes	Yes
Model 5	.3	1000	4,3,2	89%	40,10,25	Yes	Yes
Model 5	.6	300	4,2,2	73%	40,17,42	Yes	Yes
Model 5	.6	500	4,2,2	52%	40,9,22	Yes	Yes
Model 5	.6	1000	4,2,1	59%	40,6,15	Yes	Yes

Note. IFC = Interfactor correlations; *n* = sample size; NF = Number of factors in population (P), number of factors after the algorithm (A), and the number of factors in the final model (F); Replicability = Percent of subsamples replicated with that number of factors; OV = Number of observed variables in population (P), number of observed variables in the sample (S) after the FS algorithm, and percent (%) of observed variables after the FS algorithm; Model fit = Whether the final model fit the data across the four fit statistics (i.e., CFI & TLI > .95 and RMSEA and SRMR < .08).

#### Model 6

With 14 observed variables on the first factor, four observed variables on each of the other three factors, and a complex factor structure, the results (see Table 9) were mixed depending on the sample size, interfactor correlation, and method to determine the number of factors. Once again, the algorithm tended to retain more factors with smaller correlated factors (i.e., ρ = 0 and .3 conditions) and retained more observed variables as the sample size increased. For this factor structure, parallel analysis and EGA produced rather similar results when connected to the number of factors, factor replicability, percentage of observed variables retained, and model fit results.

**Table 9.** Summary of Final Factor Analysis Across Conditions for Model 6

Model	IFC	*n*	NF (P,A,F)	Replicability	OV (P,S,%)	EFA model fit	CFA model fit
*Parallel Analysis*							
Model 6	0	300	4,3,3	95%	26,15,58	Yes	No
Model 6	0	500	4,3,3	94%	26,16,62	Yes	No
Model 6	0	1000	4,3,3	91%	26,20,77	No	No
Model 6	.3	300	4,3,3	69%	26,17,65	Yes	No
Model 6	.3	500	4,3,3	92%	26,18,69	Yes	No
Model 6	.3	1000	4,3,2	70%	26,19,73	Yes	No
Model 6	.6	300	4,2,2	55%	26,22,85	No	No
Model 6	.6	500	4,2,2	92%	26,22,85	No	No
Model 6	.6	1000	4,2,2	98%	26,22,85	Yes	No
*Exploratory Graph Analysis*							
Model 6	0	300	4,3,3	90%	26,16,62	No	No
Model 6	0	500	4,3,3	96%	26,17,65	Yes	No
Model 6	0	1000	4,3,3	97%	26,20,77	No	No
Model 6	.3	300	4,3,3	73%	26,17,65	Yes	No
Model 6	.3	500	4,3,3	91%	26,18,69	Yes	No
Model 6	.3	1000	4,3,3	73%	26,18,69	Yes	No
Model 6	.6	300	4,2,1	77%	26,10,38	Yes	Yes
Model 6	.6	500	4,2,1	64%	26,13,50	Yes	Yes
Model 6	.6	1000	4,2,1	85%	26,14,54	Yes	Yes

Note. IFC = Interfactor correlations; *n* = sample size; NF = Number of factors in population (P), number of factors after the algorithm (A), and the number of factors in the final model (F); Replicability = Percent of subsamples replicated with that number of factors; OV = Number of observed variables in population (P), number of observed variables in the sample (S) after the FS algorithm, and percent (%) of observed variables after the FS algorithm; Model fit = Whether the final model fit the data across the four fit statistics (i.e., CFI & TLI > .95 and RMSEA and SRMR < .08).

Contrary to previous model results, parallel analysis retained more factors (2 vs. 1) in the final solution than EGA when the interfactor correlation was large (ρ = .6). As might be expected given the model complexity, the RFAS algorithm was more often able to retain acceptable fitting EFA models. In contrast, the only acceptable CFA model fit was for ρ = .6 with EGA when there was a single final factor solution. Given the model complexity and the smaller number of observed variables on three of the four factors, these algorithm results using the default settings are not unexpected. Consequently, the algorithm results would provide the following suggestions: (a) allow for a more complex factor structure and avoid using restrictive CFA models, (b) revise or add observed variables (e.g., items) that are a better measure of the latent factor, and/or (c) identify (and perhaps retain) the set of observed variables that are most likely to be retained in future analyses, even if they do not meet the 80% rule.

### Study 1. Modified Algorithm Results

Across many of the model conditions examined, the default settings did not perform optimally for the data structure. In several cases, the algorithm prioritized a simple structure even when the true factor structure was more complex, or it removed a substantial proportion of observed variables and/or factors. As a result, fewer replicable factor structures emerged, and these were often highly dependent on the sequence in which variables were eliminated. Such instability is a common challenge in applied research, particularly when factor structures are compared across studies or analysts.

Although the algorithm defaults often provide a good starting point, analysts may need to review the model results (e.g., reasons each observed variable was removed, observed variables’ replicability rating, and the final factor results) to determine what algorithm changes might be required to meet the desired outcome. As an example, the analyst might be willing to allow for a more complex factor structure with more observed variables and factors if it is replicable. When doing this, the analyst needs to conduct a CFA outside of the RFAS algorithm and estimate the cross-loadings, given that the RFAS is currently only programmed to estimate pure simple structures. Here, several conditions (see Table 10) that performed poorly with the default settings were reevaluated using alternative algorithm settings to reexamine the final factor solution.

**Table 10.** Summary of Final Factor Analysis Across Conditions for Default and Modified Algorithm Settings

Model	IFC	*n*	NF (P,A,F)	Replicability	OV (P,S,%)	EFA model fit	CFA model fit
Model 2 Defaults (PA)	.3	300	4,4,3	60%	40,16,40	No	No
Model 2 Modified (PA)	.3	300	4,4,4	79%	40,29,73	Yes	No
Model 2 Defaults (EGA)	.6	1000	4,3,3	73%	40,19,48	Yes	Yes
Model 2 Modified (EGA)	.6	1000	4,4,4	57%	40,27,68	Yes	No
Model 4 Defaults (PA)	0	300	4,2,2	54%	20,6,30	Yes	Yes
Model 4 Modified (PA)	0	300	4,3,3	46%	20,10,50	Yes	No
Model 5 Defaults (EGA)	.6	500	4,2,2	52%	40,9,22	Yes	Yes
Model 5 Modified (EGA)	.6	500	4,3,3	60%	40,22,55	Yes	Yes
Model 6 Defaults (EGA)	.6	500	4,2,1	64%	26,13,50	Yes	Yes
Model 6 Modified (EGA)	.6	500	4,3,3	84%	26,22,85	Yes	Yes

Note. IFC = Interfactor correlations; *n* = sample size; NF = Number of factors in population (P), number of factors after the algorithm (A), and the number of factors in the final model (F); Replicability = Percent of subsamples replicated with that number of factors; OV = Number of observed variables in population (P), number of observed variables in the sample (S) after the FS algorithm, and percent (%) of observed variables after the FS algorithm; Model fit = Whether the final model fit the data across the four fit statistics (i.e., CFI & TLI > .95 and RMSEA and SRMR < .08).

The first condition re-examined was Model 2 (*r* = .3, *n* = 300) with parallel analysis. In this case, the factor replicability rating was low (i.e., 60%) and a larger percentage (i.e., 60%) of the observed variables were removed. After examining the reason, each observed variable was removed, the observed variable’s replicability rating, and the final algorithm solution, the algorithm settings were changed to a D-score of 1.5 and a cut value (i.e., observed variables replicability rating or the proportion of samples an observed variable needs to be in the final solution) of .6 to allow for more model complexity and the retainment of more observed variables, respectively.

Following these changes, a four-factor solution was obtained with 73% of observed variables being retained and the factor replicability rating increased to 79%. Although the EFA fit the data well (CFI = .99, TLI = .99, RMSEA = .02, SRMR = .04), the CFA model fit (CFI = .85, TLI = .84, RMSEA = .06, SRMR = .01) was rather poor. However, these CFA model fit results were not entirely unexpected given the more complex factor structure (recall that all CFA secondary loadings were fixed at zero) and the small sample size (*n* = 102). Despite the improved model performance, these results point to concerns associated with using smaller sample sizes (both for the training and validation samples) and the fact that analysts should not always expect the CFA models to fit the data with complex factor structures.

The second condition reexamined was Model 2 (*r* = .6, *n* = 1000) with EGA. In this case, the replicability rating was high (73%) using the algorithm defaults, but a larger percentage (52%) of the observed variables was removed. Although one could certainly argue this is an acceptable solution (i.e., a replicable 3-factor solution with 19 observed variables that fit the EFA and CFA models well), an alternative solution was explored to increase the number of observed variables remaining. Here, the only algorithm setting changed was the D-score to two (rather than three) to allow for increased model complexity.

The revised algorithm results now suggest a 4-factor solution with 27 (so 68% retained) observed variables, but the replicability rating decreased to 57% and two of the CFA model statistics (i.e., CFI and TLI) were slightly less than the .95 criteria (CFI = .94, TLI = .94, RMSEA = .05, SRMR = .06). In this case, the analyst needs to decide if they prefer a more replicable (i.e., 73%) factor structure with 3-factors and 19 observed variables or a less replicable (i.e., 57%) factor structure with 4-factors and 27 observed variables.

The RFAS algorithm could, in principle, be revised to evaluate alternative solutions. Although neither solution examined here can be considered ideal, the findings highlight an important conclusion: Not all factor structures are easily replicable across different samples. Future researchers should take this limitation into account and consider modifying the set of observed variables, such as revising item statements or introducing new items, to enhance the replicability of factor analytic results.

Model 4 (*r* = 0, *n* = 300) with parallel analysis was also reexamined to determine an alternative factor solution. In this case, the algorithm setting changes were cut = .60, primary factor loading = .35, and D-score = 1.5. The lower cut value allows a greater number of observed variables to remain in the final solution (i.e., rather than an observed variable having to remain in 80% of the solutions, it only had to remain in 60%). The lower primary factor loading value allowed for smaller primary loadings (i.e., λ≥ .|35| rather than λ≥ .|40|) to remain in the final solution. The smaller D-score allowed the primary factor loading to be only 1.5 times larger than the variance attributed to the secondary factor (i.e., it allowed for a larger collective secondary loadings).

These algorithm modifications produced an additional factor and four additional (or 20% increase) observed variables. At the cost of these additional observed variables and factors, the replicability rating decreased by 8% and the CFA model fit (CFI = .95, TLI = .93, RMSEA = .04, SRMR = .07) was slightly below the standards. Similar to many factor analysis studies, these results point to the concerns associated with obtaining a replicable factor solution with complex factor structures and smaller sample sizes. These results also point to the conclusion that either a larger sample size is needed, or the set of observed variables should be revised if future research can hope for replicable factor solutions.

An alternative model with EGA was also examined for the Model 5 (*r* = .6, *n* = 500) condition. For this algorithm run, the cut value was reduced from .8 to .6 to permit a greater number of observed variables, and the D-score was decreased to 1.5 to allow for more model complexity. These changes resulted in an additional factor, an additional 13 observed variables (or 33% increase), and an increased replicability rating of 8%. In this case, revising the algorithm setting appeared to produce a more desirable factor solution. However, the analyst would be required to conduct a deeper examination of this solution to determine whether it is acceptable. This type of deeper examination was conducted for the revised Model 6 (*r* = .6, *n* = 500) condition below.

In addition to the summary results (see Table 10) provided from the modified RFAS algorithm settings for the Model 6 (*r* = .6, *n* = 500) condition with EGA, the EFA factor loadings and observed variable replicability ratings were also examined and reported (see Table 11). For this run, the cut value was reduced from .8 to .6 to retain a greater number of observed variables in the final solution, and the D-score was decreased to 1.5 to allow for more model complexity. The percentage of training data was increased from 66% to 70% to allocate a slightly larger percentage for the EFA analyses. These changes resulted in two additional factors, nine additional observed variables (35% increase), and an increased replicability rating of 20% (i.e., from 64 to 84).

**Table 11.** Provides the EFA Factor Loadings for Model 6 (*r* = .6, *n* = 500) Using the Default and Modified Algorithm Settings

Variable	Default settings		Variable	Modified settings			
	F1	OVR		F1	F2	F3	OVR
V1	**0.74**	0.94	V1	**0.60**	**0.24**	0.01	1.00
V2	**0.82**	0.98	V2	**0.72**	**0.20**	0.03	1.00
V3	**0.72**	1.00	V3	**0.57**	0.16	-0.08	1.00
V4	**0.70**	1.00	V4	**0.62**	0.09	0.00	1.00
V5	**0.71**	1.00	V5	**0.76**	0.09	0.14	1.00
V6	**0.76**	1.00	V6	**0.70**	-0.02	-0.18	1.00
V7	**0.64**	0.97	V7	**0.81**	-0.15	0.06	1.00
V8	**0.68**	1.00	V8	**0.71**	0.00	-0.02	1.00
V9	**0.69**	0.84	V9	**0.58**	**0.24**	0.05	1.00
V10	**0.83**	0.83	V10	**0.60**	**0.23**	-0.13	1.00
V11	**0.68**	1.00	V11	**0.67**	-0.04	-0.15	1.00
V12	**0.77**	1.00	V12	**0.82**	-0.07	-0.10	1.00
V14	**0.77**	1.00	V13	**0.66**	0.07	**-0.23**	1.00
			V14	**0.68**	-0.01	**-0.21**	1.00
			V18	**0.32**	0.04	**-0.55**	0.96
			V19	-0.01	**0.73**	-0.15	1.00
			V20	0.13	**0.72**	-0.14	1.00
			V21	**0.23**	**0.66**	0.03	0.98
			V23	-0.03	**0.31**	**-0.58**	1.00
			V24	0.00	0.03	**-0.83**	1.00
			V25	**0.28**	-0.01	**-0.72**	1.00
			V26	0.07	**0.30**	**-0.62**	0.95

Note, factor loadings ≥ .|20| are bolded. OVR = Observed Variable Replicability rating, meaning the percent of time that an observed variable loaded on the final factor model within each the 100 subsamples.

The EFA factor loadings under the default and modified RFAS algorithm conditions are also provided in Table 11. The default algorithm settings produced a single factor with factor loadings between .64 and .83 and acceptable average EFA model fit statistics (CFI = .99, TLI = .99, SRMR = 0.03, RMSEA = 0.02). After modifying the algorithm settings and producing a 3-factor solution, the interfactor correlations ranged between .|52| to .|67| (*r*_12_ = .67, *r*_13_ = –.60, & *r*_23_ = .52) with acceptable average EFA model fit statistics (CFI = .99, TLI = .99, SRMR = 0.03, RMSEA = 0.02).

By relaxing the algorithm constraints, the final factor solution exhibited slightly increased model complexity. For example, V18 had larger factor loadings on factor 1 (λ = .32) and factor 3 (λ = -.55), V23 had larger factor loadings on factor 2 (λ = .31) and factor 3 (λ = -.58), and V26 had larger factor loadings on factor 1 (λ = .30) and factor 3 (λ = -.62). Despite the increased model complexity, the replicability rating for each observed variable was above .90.

From these results, it becomes evident why a replicable pure simple structure solution was difficult to obtain and, therefore, a single factor was deemed best under the RFAS default settings. Relatedly, it is worth pointing out that although the CFA model fit the data well using the modified algorithm settings (see Table 10) and the factor loadings were all large (λ > .60), multicollinearity was a significant concern for this model (*r*_12_ = .82, *r*_13_ = .83, & *r*_23_ = .78). For analysts that desire this more replicable complex structure under the modified algorithm conditions, either a modified CFA model is required (i.e., large cross-loadings estimated) or analysts would likely benefit from an exploratory structure equation model (ESEM; Asparouhov & Muthén, 2009) when estimating the structural coefficients.

In summary, the algorithm results provide considerable evidence for the factor structure’s credibility across several replications, along with guidance related to how the structure should be modified in the future. While an analyst might be able to obtain a pure, simple structure with this data based on an EFA and/or CFA model after removing a given subset of observed variables, the fact is that the subset of observed variables and factors would likely be challenging to replicate in practice and would only cause confusion and conflict between researchers.

### Study 2. Algorithm Comparison

Although the factor structures, particularly their complexity (i.e., magnitude of cross-loadings) and interfactor correlations, varied considerably across the 10 experimental conditions, both EFA and CFA generally produced acceptable model fit indices (see Table 3) with the complete variable set. EFA models consistently demonstrated excellent fit, as the larger cross-loadings could be freely estimated without adversely affecting fit statistics. In contrast, CFA models occasionally exhibited suboptimal fit, primarily due to increased model complexity and the constraint of fixing these cross-loadings to zero.

For the ACO method, the mean Jaccard similarity coefficients ranged from .408 to .835 across the ten model conditions (see Table 3). Models 1, 4, and 6 demonstrated the highest stability, indicating acceptable agreement in the observed variables retained across replications. In contrast, Models 2 and 5 showed lower stability, suggesting greater variability in the selected variable sets. These results suggest that variable selection stability is highly sensitive to data and model specifications, including factor structure complexity, sample size, and the targeted number of observed variables per factor. Overall, these results are somewhat concerning. For example, even with a large sample size and a clean factor structure (e.g., Model 1 [0.3, 1000, 40]), the subset of observed variables retained could vary drastically across subsamples, leaving the analyst perplexed about which subset of variables to retain in the final model. With more complex factor structures (e.g., Models 2 & 5), these results were even more concerning, leading researchers to be perplexed about the best variable subset.

Table 12 presents the percentage of retained observed variables for each model, with bold values indicating observed variables retained in at least 80% of the subsamples. Across the 20 model conditions (10 ACO & 10 RFAS), the proportion of variables exceeding the 80% stability threshold varied substantially, ranging from as low as 3% to as high as 100%. RFAS generally retained a higher proportion of variables above the 80% threshold compared to ACO, with RFAS proportions ranging from 28% (Model 5, 0.6, 500) to 100% (Model 6, 0.3, 500) and ACO proportions ranging from 3% (Model 5, 0.6, 500) to 85% (Model 6, 0.6, 500). In several conditions, particularly Model 1 (0.3, 1000), Model 2 (0.3, 500), Model 6 (0.3, 500), and Model 6 (0.6, 500), RFAS achieved markedly higher reproducibility rates, with over two-thirds of items retained in at least 80% of subsamples. RFAS also retained a significantly higher percentage of variables above 80% (M = 67.70, *SD* = 22.93) compared to ACO (M = 34.30, *SD* = 27.73), *t* (18) = 2.935, *p* = .009, d = 1.31.

**Table 12.** Percentage of Retained Variables Per Model, Along With the Percentages of Variables Retained in Over 80% of the Subsamples (Bolded)

	M1 (0.3, 1000)		M1 (0.6, 300)		M2 (0.3, 500)		M2 (0.6, 1000)		M3 (0.6, 1000)		M4 (0.3, 1000)		M5 (0.6, 500)		M5 (0.3, 1000)		M6 (0.3, 500)		M6 (0.6, 500)	
Item	RFAS	ACO	RFAS	ACO	RFAS	ACO	RFAS	ACO	RFAS	ACO	RFAS	ACO	RFAS	ACO	RFAS	ACO	RFAS	ACO	RFAS	ACO
X1	**100**	**88**	**89**	**85**	**100**	55	**100**	49	**100**	42	**100**	5	21	33	0	13	68	62	**100**	74
X2	**99**	43	44	52	7	8	14	17	**100**	**86**	0	**99**	77	26	**99**	29	**98**	**100**	**100**	**98**
X3	**100**	54	**99**	69	33	65	**81**	74	**100**	**81**	**100**	**99**	83	39	**99**	50	**98**	68	**100**	70
X4	**100**	79	32	73	17	12	**81**	13	**99**	19	**100**	**97**	31	75	**95**	66	**100**	32	**100**	30
X5	**100**	**91**	**95**	**92**	**100**	72	**100**	75	**100**	72	**98**	**100**	42	34	**82**	47	**100**	79	**100**	65
X6	69	30	8	45	**82**	22	**91**	21	**100**	17	**100**	**100**	31	75	49	**84**	**100**	**97**	**100**	**92**
X7	**100**	**82**	**99**	72	44	79	**96**	**82**	**100**	59	**100**	**98**	52	59	**92**	73	**100**	49	**100**	12
X8	**100**	79	**99**	66	28	71	63	**81**	**100**	36	**100**	**95**	29	70	56	73	**100**	57	**100**	46
X9	**100**	**86**	**98**	79	**99**	64	**100**	54	**100**	32	**100**	**100**	21	73	**83**	62	70	37	**100**	59
X10	**100**	68	**94**	67	72	47	**98**	34	**100**	77	15	7	46	16	49	3	71	**91**	**100**	**98**
X11	44	28	27	37	**87**	30	25	29	**100**	33	1	**99**	26	32	**97**	15	**100**	59	**100**	71
X12	**100**	55	72	57	**89**	36	70	31	**100**	80	0	79	29	66	3	75	**100**	**93**	**100**	**96**
X13	**99**	41	31	35	**99**	74	**96**	60	**100**	64	30	72	4	4	**93**	2	**90**	**90**	**100**	**97**
X14	**100**	**95**	**100**	**85**	**95**	32	**100**	23	**100**	13	2	**99**	62	45	3	63	**100**	**86**	**100**	**92**
X15	**100**	57	**100**	44	63	59	**100**	48	**97**	**100**	0	51	16	48	**97**	30	8	**93**	57	**95**
X16	**100**	**91**	**100**	**95**	0	43	0	**82**	**100**	**100**	49	**100**	12	**83**	3	**85**	16	20	27	7
X17	**100**	**84**	**100**	**82**	**88**	77	**89**	**84**	**100**	**89**	**100**	**89**	50	67	3	74	1	**92**	56	**99**
X18	**100**	**89**	**100**	**90**	**80**	21	32	16	**100**	**98**	**83**	**96**	2	46	3	39	**91**	**95**	**96**	**99**
X19	**100**	**94**	**100**	**98**	**98**	**85**	**86**	76	**100**	66	71	69	15	78	3	76	**100**	79	**100**	71
X20	**100**	66	**80**	77	0	38	0	51	**100**	49	**100**	46	8	31	**97**	41	**100**	**100**	**100**	**96**
X21	**100**	**93**	77	**95**	**98**	43	**100**	45	**100**	64			73	36	3	48	**98**	**85**	**98**	64
X22	**100**	**90**	**100**	**84**	**85**	50	**89**	44	**100**	78			**90**	67	5	68	**85**	36	51	69
X23	**100**	41	**95**	37	38	25	**82**	28	65	38			75	30	9	18	54	25	**100**	35
X24	**100**	**86**	**95**	**91**	6	75	9	**81**	7	35			**86**	72	8	62	**100**	**98**	**99**	78
X25	**100**	45	**97**	50	40	11	**98**	14	10	31			**94**	14	9	14	**98**	**99**	**100**	**98**
X26	**100**	79	**100**	**81**	**99**	65	**100**	59	44	41			**90**	61	6	53	46	78	**95**	**89**
X27	**100**	62	**96**	59	31	50	44	43					**94**	62	8	72				
X28	**88**	26	**87**	23	**89**	17	67	30					**82**	41	9	53				
X29	**100**	79	**84**	**84**	**96**	**83**	58	**83**					**90**	68	2	67				
X30	**100**	**99**	**99**	**96**	**83**	76	**91**	73					**92**	49	7	45				
X31	**96**	57	**87**	73	46	19	0	9					**83**	69	**97**	74				
X32	**100**	76	**84**	**84**	**99**	31	**91**	21					69	61	61	48				
X33	**100**	68	**96**	62	14	57	46	78					4	30	1	69				
X34	**100**	62	**98**	54	**100**	65	**100**	56					73	75	**94**	69				
X35	78	62	48	62	**100**	42	**100**	66					71	52	3	20				
X36	**100**	79	**100**	**85**	**99**	79	**98**	**83**					75	49	**93**	32				
X37	**100**	**92**	**100**	**93**	**100**	43	**100**	31					2	27	1	63				
X38	**100**	**89**	**100**	**92**	**93**	64	**100**	59					65	47	77	54				
X39	**96**	28	41	14	**86**	32	**100**	30					81	29	**97**	17				
X40	**100**	**87**	**100**	**81**	**100**	63	**100**	67					73	61	**96**	54				
% > 80	93%	40%	78%	45%	63%	5%	68%	18%	85%	27%	55%	65%	28%	3%	38%	5%	69%	50%	100%	85%

The higher variability in retention (with more than 80% of subsamples) and the lower reproducibility may be partly attributable to the ACO setting, specifying the number of observed variables per factor. For example, if the observed variables per factor in Model 1 (0.3, 1000) were increased from 7 to 8, the percentage of variables retained (i.e., percentage of variables with greater than 80% retained) would significantly increase (from 40% to 62.5%) due to the algorithm settings.

Correlations between the RFAS and ACO solutions were examined across the 10 experimental conditions to evaluate the degree of agreement in variable selection. The correlations ranged from –.20 to .53, reflecting substantial variability in consistency between the two methods. The strongest associations were observed in the nearly pure simple structure conditions (i.e., M1 [0.3, 1000], *r* = .52, and M1 [0.6, 300], *r* = .53), both indicating moderate agreement in the subsets of observed variables retained. More modest positive correlations were found in M2 (0.3, 500) (*r* = .25) and M3 (0.6, 1000) (*r* = .33), which represented an approximate simple structure.

By contrast, correlations for M4 (0.3, 1000) (*r* = .12), M5 (0.6, 500) (*r* = .09), M6 (0.3, 500) (*r* = .19), and M6 (0.6, 500) (*r* = .21) were very weak associations, suggesting limited overlaps in variable retention across algorithms for more complex factor structure. M2 (0.6, 1000) (*r* = .08) demonstrated virtually no agreement between algorithms, while M5 (0.3, 1000) (*r* = –.20) indicated a negative association.

Although differences in algorithmic results may appear concerning, it is essential to recognize that RFAS and ACO are designed with distinct objectives. The variability in correlations observed across conditions highlights the importance of employing both methods when evaluating factor structures. Because the two algorithms often diverged, instances of convergence are particularly compelling, providing strong evidence of replicability and greater confidence that the solution is not merely an artifact of a single algorithm’s assumptions or parameter settings. Thus, the combined use of RFAS and ACO offers a more robust strategy for identifying stable and generalizable factor structures, with cross-method agreement serving as a reliable indicator of solution validity. Even when differences emerge, the comparison remains informative, as it highlights which observed variables demonstrate consistent reproducibility and prompts researchers to weigh trade-offs among outcomes such as model fit, factor structure complexity, variable retention, and replicability in determining the most appropriate solution.

## Discussion

### Study 1

Study 1 simulations evaluated the performance of the RFAS algorithm across varying factor structures, sample sizes, interfactor correlations, and algorithm settings. Results showed that replicable factor structures can be obtained with the default settings when data approximate simple structures, sample sizes are moderate to large, and interfactor correlations are low to moderate. As with other variable selection methods, adjusting the settings can yield different solutions (e.g., fewer observed variables, purer simple structures, or improved model fit), allowing analysts to tailor outcomes to their definition of an acceptable solution. These findings underscore the importance of modifying default settings when model performance is suboptimal and of assessing the sensitivity of results to different criteria.

Building on the above summary, this study revealed several notable trends. First, the number of observed variables retained generally increased with larger sample sizes, likely due to smaller estimated factor loading standard errors, which increased the probability that loading consistently exceeded or fell below the algorithm’s thresholds (i.e., for primary or secondary loading magnitude). Second, an observed variable’s replicability rating was negatively associated with both model complexity and the magnitude of interfactor correlations, as cleaner solutions that are more distinct are easier to reproduce.

Consistent with prior research (Golino et al., 2020; Golino & Epskamp, 2017), the EGA method performed as well as, or better than, parallel analysis with highly correlated factors. These findings suggest that EGA should be considered, or at minimum compared with parallel analysis, in these contexts. Although identifying the optimal method for determining the “correct” number of factors was not the primary aim of this study, the results provide reasonable evidence in favor of EGA. Nonetheless, further research is needed, and in the interim, analysts are advised to evaluate models using both approaches.

As with other variable selection algorithms, it is essential that users avoid adopting the final solution produced by RFAS without careful evaluation. Sensitivity analyses (Heinze et al., 2018), consideration of content validity, and the exploration of alternative models (e.g., subsets of highly replicated observed variables) are all recommended to ensure robust conclusions. Because changes to the algorithm settings can yield different final solutions, analysts should exercise caution when selecting these criteria and explicitly consider their implications for replicability. In particular, researchers are encouraged to evaluate alternative specifications (e.g., thresholds for primary and secondary loadings) while recognizing the potential trade-offs for both EFA and CFA outcomes. For instance, permitting larger secondary loadings may reduce the likelihood of eliminating a large number of observed variables, but this adjustment often comes at the cost of likely poorer CFA model fit (assuming cross-loadings are fixed at zero, as is the case with the RFAS algorithm) and/or inflated interfactor correlations (Sass & Schmitt, 2010).

When the algorithm does not yield an acceptable factor structure, analysts must carefully balance the relative importance of replicability, structural simplicity, and the number of retained observed variables or factors. The results can then be used to guide modifications to the default settings, tailoring model performance to the analyst’s priorities. Importantly, even factor solutions deemed unacceptable, whether derived from default or adjusted settings, can provide meaningful insights. Such outcomes may illuminate why prior studies have reported inconsistent factor structures and the next steps to improve the measurement model (e.g., change items, response scales, or the number of factors).

### Study 2

ACO results revealed notable variability in reproducibility across model conditions, suggesting that researchers may not always have confidence in the best subset of variables to retain. Therefore, researchers may need to run the model under different search criteria to find a reproducible and justifiable variable subset.

Variable retention rates further illustrate key differences between the RFAS algorithm and ACO. Across the models tested, the percentage of variables retained in ≥80% of subsamples ranged from 28% to 100% for RFAS and from 3% to 85% for ACO. RFAS consistently achieved a higher mean retention above the 80% threshold than ACO. One reason for this is that RFAS does not set any criteria on how many variables should be retained. Instead, it is based on whether each variable meets the inclusion criteria. Therefore, if seven observed variables were retained in the final model, but only five were desired, the researcher would have to decide what metrics should be used to remove two additional observed variables (e.g., it could be the reproducibility statistic, factor loading size, or impact on model fit).

It is important to note that RFAS and ACO differ in their underlying optimization objectives. The RFAS algorithm is designed to prioritize obtaining a clean, well-defined factor structure that both fits the data and retains as many variables as possible, thereby supporting broad content coverage while minimizing cross-loadings and structural complexity. In contrast, the ACO approach is configured to search for the subset of variables that best satisfies predetermined model fit criteria, with less direct emphasis on achieving a pure simple structure.

Although ACO can be adapted to incorporate penalties for complexity or cross-loadings, its default configuration in this study emphasized maximizing fit within variable-per-factor constraints rather than explicitly optimizing for structural simplicity. As a result, RFAS inherently targets structural clarity alongside fit, whereas ACO primarily optimizes for fit quality, which may result in factor solutions that are statistically acceptable but less strictly aligned with the principles of simple structure.

### Algorithm Uses

Algorithm results (regardless of algorithm and settings) could also be used for more advisory and exploratory purposes. For instance, RFAS and ACO users can utilize both preliminary algorithm results (e.g., the percentage of time each observed variable was retained) and final algorithm model results to inform their decision on which subset of variables and factors to retain for the final factor model. In other words, the algorithm results could simply be used as an informative tool to aid analysts in selecting their own final model.

Model comparison is a central component of exploratory factor analysis (EFA), with analysts encouraged to evaluate solutions that vary in the number of factors and sets of observed variables. As Tukey (1977) described, exploratory data analysis is a form of “detective work,” requiring careful consideration of multiple plausible interpretations of the same data. Algorithms are designed to facilitate this process by generating numerous potential factor solutions and providing information on both structural quality (e.g., loadings, complexity) and replicability. For instance, the RFAS algorithm estimates thousands of EFA models and enables analysts to assess the appropriate number of factors and observed variables to retain based on patterns emerging across these replications.

### Algorithm Considerations

An objective, algorithmic approach to building factor models offers clear benefits, particularly in enhancing replicability across studies and producing more generalizable models and statistical results. At the same time, there is a compelling case for allowing a degree of subjectivity in model construction. Theoretical considerations may justify the inclusion of certain observed variables that a purely mechanistic algorithm would exclude. Put differently, while algorithms may prioritize statistical fit and simplicity, they risk discarding variables essential for adequately representing a construct, thereby undermining content validity and increasing the likelihood of construct underrepresentation. Thus, algorithms should be viewed not as prescriptive but as informative, shedding light on the reproducibility of factor models and supporting analysts in balancing statistical evidence with conceptual priorities. In the context of instrument development and validation, traditional concerns with validity and reliability remain essential, and algorithms, like RFAS, were designed to enhance transparency and reinforce sound factor-analytic practice.

### Limitations

Despite the benefits of the RFAS and ACO algorithms, several limitations should be noted. First, there is no guarantee that either algorithm will identify the “best variable subset,” or will they converge on similar solutions. Their key strength lies in enhancing transparency: by standardizing analytic decisions and emphasizing factor structure reproducibility. In this way, algorithmic results offer insight into why factor analytic findings diverge across studies and can help reveal factor structures that are theoretically proposed but not empirically supported (Schmitt et al., 2018).

Second, analysts may differ in their priorities and criteria for what constitutes an acceptable solution. For example, some may place less emphasis on global model fit, tolerate larger secondary loadings, or accept lower replicability thresholds for variables or factors. The RFAS algorithm accommodates such flexibility by allowing users to state their decision criteria, which in turn clarifies how final solutions are generated. Importantly, RFAS provides potential solutions and replicability information, but it does not account for other forms of validity and reliability. Analysts must therefore integrate algorithmic results with sound psychometric judgment when finalizing a factor structure.

Third, both RFAS and ACO require analysts to make subjective decisions regarding algorithm settings, which introduces an element of researcher judgment into the process. For example, analysts may need to adjust inclusion thresholds, such as the minimum replicability rating or the number of variables per factor required for identification, depending on the purpose of the analysis. Similarly, choices must be made about model fit criteria, whether to prioritize strict model fit cutoffs or to adopt more flexible thresholds to retain a broader set of observed variables. In some cases, researchers may also decide to remove problematic or poorly performing items before running the algorithm, effectively pruning the model in advance to improve convergence and interpretability.

These types of decisions underscore that variable selection algorithms are not fully automated solutions but rather tools to support the analytic process. While RFAS and ACO provide systematic ways to evaluate replicability and model fit, they cannot substitute for statistical expertise, theoretical grounding, or substantive knowledge of the constructs under investigation. Analysts must balance statistical evidence with considerations of content validity, theoretical coverage, and practical utility when determining the final factor solution. In this sense, algorithmic results should be viewed as decision aids that enhance transparency and reproducibility, rather than as definitive arbiters of the “correct” model.

With respect to Study 2, a more comprehensive evaluation of variable selection algorithms for factor analysis is warranted. Future research should not only clarify the conditions under which each algorithm performs optimally but also investigate the tuning parameters that maximize their effectiveness across different data structures. Although the present findings suggested that WGL and stepwise methods rarely eliminate observed variables, this should not be interpreted as evidence that these approaches lack utility. Regardless, these methods should be explored to determine their role and value for variable selection within factor analysis.

## Conclusion

The RFAS algorithm was developed to encourage researchers to generate factor solutions that are more reproducible and grounded in sound statistical and psychometric principles. Historically, factor analytic research has varied widely in estimation methods, correlation matrices, rules for determining the number of factors, rotation strategies, and variable selection approaches. Such methodological inconsistency often produces divergent factor structures, making it difficult to determine whether differences are attributable to sampling error or analytic choices. Factor analytic algorithms, like RFAS and ACO, directly address this concern by providing systematic evidence on factor structure quality, reproducibility, and stability. In doing so, they offer researchers a transparent and rigorous framework for evaluating factor solutions and strengthen the foundations for cumulative knowledge in applied psychometric research.

## Notes

1. These results are provided by the RFAS algorithm.

2. The Mplus code used for data simulation and the full RFAS algorithm are provided on the RFAS website (www.RFASalgorithm.com).

3. While the simulated interfactor population correlations were .60, the correlations between the rotated population correlations were higher .69 ≥ρ≤ .79.
